# Local Societies

**Published:** 1882-11

**Authors:** 


### ARTICLE VIII.

### LOCAL SOCIETIES.

After a rather long vacation, the Biological Society resumed its monthly meetings, October 4, in Doctor P. S. Hayes' office, 167 Wabash avenue. It is to be deplored that the Tremont could no longer shelter this scientific body. The new location cannot be more than a temporary one as the want of sufficient room may be felt any time when the Society turns out in full.

This want of the proper locations and accommodation for our various medical societies has long been felt and never but partially remedied. It is nearly two years since the Pathological Society moved its quarters from the Washingtonian Home to 533 W. Adams street, the residence of their founder and actual president, Dr. H. M. Lyman. This new departure proves very successful, mostly on account of the amenity of Dr. Lyman and the amplitude of his rooms. His suggestion that the Cook County Hospital might at a future date offer a good place wherein the various societies could meet, is especially worthy of consideration now that the Hospital is being enlarged and new county commissioners elected. But why do not the various medical colleges offer meeting rooms for our various medical societies?

Strange, but a glance will show that the city of Chicago is wonderfully deficient in scientific societies. The Academy of Sciences after a manly struggle with adverse circumstances, has finally been forced to sell its building to liquidate a large a indebtedness, and the *only museum in Chicago* is in as disordered a condition almost as the breccia of the cave of Engis when excavated by Schmerling; all for want of room. It seems that before selling their property an attempt had been made at selling titles, for we read in their by-laws, article xi., "Any person paying twenty-five hundred dollars into the hands of the treasurer, shall be a 'Patron of the Academy,' a life member thereof, etc." The annual fee of five dollars, and initiation fee of ten, was considered too high by many professional men. Still, the curator of the Museum lately expressed his belief that the Academy of Sciences never stood on a firmer basis.

A new society which seems especially prosperous just now is the Electrical Society. They meet the last Monday of each month at the Grand Pacific hotel. Several doctors belong to it.

The Gynæcological Society has the special merit of ending its meetings with a banquet and thus combine *l'utile et l'agréeable.* As might be expected this society is not large, and seems the infancy of what may prove a worthy adult.

The latest sensation is the formation of a society of German physicians, whose place of meeting is over a saloon on the North Side, all for the want of proper accommodations.

Not to leave anything overlooked and incur the reproach of sectarianism, we must say that our Eclectic brethren have lately been heard from. A member of their society has been rejected for compounding a patent medicine, the excluded member sued the society for libel and tried to prove, by the testimony of people who had used it, that his medicine was all that it was advertised to be. But the testimony was not accepted in court, and we felicitate the Eclectics on their narrow escape.

Perhaps the best attended society in Chicago, is the Philosophical Society, which meets Saturday evenings at Apollo Hall, (Central Music Hall). Some very valuable essays are read at these meetings, a few of them by medical men, as that by Prof. Jewell, which is on the programme for the 20th of January next. The only reproach to which this society is liable, perhaps, is a sort of offensive anti-religious tendency manifested by some members. It seems to count the élite of Chicago in its ranks.

The Chicago Medical Society met October 23, at the Grand Pacific hotel, and Dr. Roswell Park read his valuable paper on "The Present Status of Antiseptic Surgery," published in the last number of the JOURNAL, a paper too complete and masterly to need any comment; and the discussion which followed was of little import.

Dr. C. T. Parkes approved of the paper in all its details and said that owing to the amount of trouble necessary for applying Listerism or the aseptic method, he considered it preferable to resort to the common antiseptic dressings. Notwithstanding the expenses of either method, he considered that they were indispensable in all important operations. Some antiseptics were not disagreeable and should often be preferred on that account to carbolic acid. Boracic acid was valuable in that respect, and it was not liable to be followed by erysipelas. In dissection wounds, alcohol was a good fluid to saturate lint with, in dressing the parts.

Dr. E. Powell sustained this last recommendation, and insisted on the use of carbolic acid in operations in which the abdomen or the joints were opened.

The following delegates were chosen to attend the meeting of the American Public Health Association: Drs. H. A. Johnson, R. E. Starkweather, L. H. Montgomery.

